# Population-Based Active Surveillance for Culture-Confirmed Candidemia — 10 Sites, United States, 2017–2021

**DOI:** 10.15585/mmwr.ss7404a1

**Published:** 2025-05-29

**Authors:** Emily N. Jenkins, Jeremy A.W. Gold, Kaitlin Benedict, Shawn R. Lockhart, Elizabeth L. Berkow, Tamia Dixon, Shanita L. Shack, Lucy S. Witt, Lee H. Harrison, Shannon Seopaul, Maria A. Correa, Megan Fitzsimons, Yalda Jabarkhyl, Devra Barter, Christopher A. Czaja, Helen Johnston, Tiffanie Markus, William Schaffner, Annastasia Gross, Ruth Lynfield, Laura Tourdot, Joelle Nadle, Jeremy Roland, Gabriela Escutia, Alexia Y. Zhang, Anita Gellert, Christine Hurley, Brenda L. Tesini, Erin C. Phipps, Sarah Shrum Davis, Meghan Lyman

**Affiliations:** ^1^ASRT Inc., Atlanta, Georgia; ^2^Division of Foodborne, Waterborne, and Environmental Diseases, National Center for Emerging and Zoonotic Infectious Diseases, CDC, Atlanta, Georgia; ^3^Georgia Emerging Infections Program, Georgia Department of Public Health, Atlanta, Georgia; ^4^Emory University School of Medicine, Atlanta, Georgia; ^5^Atlanta Veterans Affairs Health System, Decatur, Georgia; ^6^Johns Hopkins Bloomberg School of Public Health, Baltimore, Maryland; ^7^Connecticut Emerging Infections Program, Yale School of Public Health, New Haven, Connecticut; ^8^Colorado Department of Public Health and Environment, Denver, Colorado; ^9^Vanderbilt University Medical Center, Nashville, Tennessee; ^10^Minnesota Department of Health, St. Paul, Minnesota; ^11^California Emerging Infections Program, Oakland, California; ^12^Public Health Division, Oregon Health Authority, Portland, Oregon; ^13^University of Rochester Medical Center, Rochester, New York; ^14^New Mexico Emerging Infections Program, Albuquerque, New Mexico

## Abstract

**Problem/Condition:**

Candidemia, a bloodstream infection caused by *Candida* spp., is a common cause of health care–associated bloodstream infections in the United States. Candidemia is associated with substantial health care costs, morbidity, and mortality.

**Period Covered:**

2017–2021.

**Description of System:**

CDC’s Emerging Infections Program (EIP), a collaboration among CDC, state health departments, and academic partners, was used to conduct active, population-based laboratory surveillance for candidemia at city or county sites located in 10 states (California, Colorado, Connecticut, Georgia, Maryland, Minnesota, New Mexico, New York, Oregon, and Tennessee), representing a combined population of approximately 21.5 million persons, or 7% of the U.S. population in 2019. Connecticut began reporting cases on January 1, 2019, and conducts statewide surveillance. Although candidemia is not a nationally notifiable condition, cases of *Candida auris* infection are nationally notifiable, and cases of candidemia caused by *C. auris* could be included in both national case counts and EIP surveillance. A culture-confirmed candidemia case is defined as a positive blood culture for any *Candida* sp. from a resident in the surveillance catchment area. Subsequent positive blood cultures for *Candida* within 30 days of the initial positive culture (index date) in the same patient are considered part of the same case. Clinical laboratories serving each catchment area report candidemia cases, and trained surveillance officers abstract information from medical charts for all cases. Corresponding isolates are sent to CDC for species confirmation and antifungal susceptibility testing.

**Results:**

A total of 7,381 candidemia cases were identified during the surveillance period (2017–2021). The overall incidence was 7.4 cases per 100,000 population. Across age groups, sexes, racial and ethnic groups, and surveillance sites, incidence was generally stable or increased slightly from 2017 to 2021, with the lowest overall incidence in 2019 (6.8) and the highest in 2021 (7.9). In 2021, candidemia incidence was highest in patients aged ≥65 years (22.7) and infants (aged <1 year) (8.0). Incidence was higher in males (8.7) compared with females (7.0) and higher in non-Hispanic Black or African American (Black) patients (12.8) compared with non-Black patients (5.6). Incidence was highest in Maryland (14.5), followed by Tennessee (10.1) and Georgia (10.0); incidence was lowest in Oregon (4.8). Increases occurred in the percentage of cases classified as health care onset (52.2% in 2017 to 58.0% in 2021). Overall, among 7,381 cases (in 6,235 patients), 63.7% occurred in patients who had a central venous catheter, 80.7% involved recent systemic antibiotic receipt, and 9.0% occurred in patients who had a history of injection drug use. The percentage of cases with a positive SARS-CoV-2 test during the 90 days before or after the index date increased from 10.4% in 2020 to 17.7% in 2021. From 2017 to 2021, the percentage of cases involving an intensive care unit stay before the index date increased from 38.3% to 44.9%. Echinocandins (e.g., micafungin) were used as treatment in 49.8% of cases, and azoles were used in 47.7%. The all-cause in-hospital mortality rate was 32.6%; this increased from 26.8% in 2019 to 36.1% in 2021. Overall, *Candida albicans* accounted for 37.1% of cases, followed by *Candida glabrata* (30.4%) and *Candida parapsilosis* (13.5%); however, *C. glabrata* was the most frequent species in California (38.4%) and Maryland (32.9%). *Candida auris* infections accounted for 0.4% of cases. Among 6,576 *Candida* isolates for which interpretive breakpoints exist and isolates were available for testing, 5.6% were fluconazole resistant, and <1% were echinocandin resistant. Antifungal resistance was stable for all antifungals tested across years.

**Interpretation:**

Candidemia remains an important health care–associated infection. The disproportionate incidence among older adults, males, and Black patients is consistent with previous reports, and the overall incidence of candidemia has not changed substantially compared with previous EIP findings based on data collected during 2012–2016 (8.7 per 100,000 population). The higher mortality rate associated with candidemia during 2020–2021 likely reflects consequences of the COVID-19 pandemic, including strained health care systems and an increased population of patients who were susceptible to candidemia because of COVID-19–related critical illness.

**Public Health Action:**

Strict implementation of measures to prevent health care–associated bloodstream infections is important to help prevent candidemia cases. Health care officials and providers should be vigilant for candidemia as a complication of critical illness. Continued surveillance is needed to monitor for emerging populations at risk for candidemia and changes in antifungal resistance patterns, which can help guide antifungal treatment selection.

## Introduction

Candidiasis is a fungal infection caused by yeast from the genus *Candida* (*1*). *Candida* spp. are a normal component of the gastrointestinal microbiome and can colonize the skin. Candidiasis can be mucocutaneous or invasive; the most common invasive form is *Candida* bloodstream infection (candidemia), which is among the most frequent health care–associated infections in the United States (*2*). Additional forms of invasive candidiasis can occur after dissemination of *Candida* from the bloodstream to other normally sterile body sites (e.g., the abdomen, bones, eyes, heart, kidneys, or lungs). Candidemia is associated with costly hospitalizations, high morbidity, and high all-cause mortality (approximately 25%) (*3*–*5*).

Candidemia can occur after disruption of the body’s skin and mucosal barriers, including in the intestines (*6*). The infection might develop after translocation of *Candida* spp. from the gut through disruptions in the intestinal mucosa, making abdominal surgeries a candidemia risk factor (*7*). Critical illness might predispose patients to candidemia because of immune dysregulation from physiologic stress and because care for critically ill patients often involves using indwelling medical devices (e.g., central venous catheters), which can serve as entry points for infection. Additional candidemia risk factors include malignancies, hemodialysis, diabetes, and receipt of immunosuppressive medications (including corticosteroids); total parenteral nutrition; and systemic antibacterial medications (*7*). Injection drug use was more recently identified as an important risk factor for non-health care–associated candidemia (*8*–*10*).

Historically, *Candida albicans* has been the leading cause of candidemia; however, candidemia involving non-*C.* *albicans* spp., especially *Candida glabrata*, *Candida parapsilosis*, *Candida tropicalis*, and *Candida krusei,* has become more prevalent in recent years (*11*). Compared with *C. albicans*, these species more frequently exhibit antifungal resistance and might be associated with higher mortality rates for patients with candidemia (*12*–*14*). In addition, *Candida auris*, a highly transmissible, and frequently multidrug-resistant *Candida* sp., has emerged as a cause of health care–associated outbreaks and candidemia (*15*,*16*).

Population-based surveillance in two U.S. metropolitan areas found that annual candidemia incidence per 100,000 population declined during 2008–2013 (from 14.1 to 9.5 in Atlanta and 30.9 to 14.4 in Baltimore), likely because of improved infection prevention and control practices (*17*). Candidemia incidence across four U.S. surveillance sites remained steady through 2016, at approximately nine cases per 100,000 population, with incidence varying by age, sex, racial and ethnic group, and geography (*3*). Analyses of 2019–2021 large health care claims and vital statistics data found an increase in the rates of hospitalizations and deaths caused by fungal infections, including invasive candidiasis (*18*,*19*), potentially related to a COVID-19–related increase in patients with critical illness and limitations in enforcement of infection prevention and control measures (*20*).

Candidemia surveillance, conducted through CDC’s Emerging Infections Program (EIP), monitors changes in U.S. candidemia epidemiology (*3*). The candidemia surveillance program began at two sites in 2008 and has since expanded to 10 sites. Updated information about demographic features, underlying conditions, treatment practices, and resistance can help guide candidemia prevention and management efforts. For this report, 2017–2021 EIP data from 10 sites were analyzed to describe candidemia incidence and characterize changes in risk factors, treatment, and outcomes over time. Health care providers, public health departments, and researchers can use this information to help inform diagnostic and treatment practices and refine clinical guidelines.

## Methods

### Data Source

Because candidemia is not a nationally notifiable condition, CDC conducts population-based active surveillance for culture-confirmed candidemia through EIP in specific counties in 10 U.S. states. Nine of these sites continuously reported surveillance data during 2017–2021, including California (Alameda County), Colorado (five counties in the metropolitan Denver area), Georgia (eight counties in the metropolitan Atlanta area), Maryland (city of Baltimore and Baltimore County), Minnesota (seven counties in the Minneapolis–St. Paul area), New Mexico (Bernalillo County), New York (Monroe County), Oregon (Portland tri-county area), and Tennessee (nine counties surrounding Knoxville in east Tennessee). The remaining site, Connecticut (all counties), began statewide candidemia surveillance on January 1, 2019. The combined population under surveillance was approximately 21.5 million persons, representing approximately 7% of the U.S. population in 2019. Cases of *C. auris* infection are nationally notifiable, and cases of candidemia caused by *C. auris* could be included in both national case counts and EIP surveillance.

### Surveillance Case Definition

A culture-confirmed candidemia case was defined as a positive blood culture for any *Candida* sp. from a resident in the surveillance catchment area. A positive *Candida* culture within the 30-day period after the initial blood culture in the same person was considered part of the same case; a positive *Candida* culture after the 30-day period in the same person was considered a new case. The 30-day period was calculated using the specimen collection date for the initial positive *Candida* blood culture, referred to as the index date. Unless noted, data in this report are presented at the case level because exposure variables can vary across cases in the same person. However, only demographic data collected during the patient’s first case of candidemia are presented for patients who had more than one case.

### Data Collection

Candidemia cases were identified through clinical, reference, and commercial laboratories serving the surveillance areas and were reported to the local surveillance officers at each site. For each case, trained surveillance officers from EIP sites reviewed and completed a standardized case report form using information from electronic medical records; the case report form included information on demographics, underlying health conditions, health care use, treatment, and outcomes. Certain data elements were not collected every year; therefore, analyses of these data elements are limited to the years with data available. In 2020, supplemental questions were added to the case report form to collect information about COVID-19 and related risk factors.

Cases were classified as “community onset” if the initial positive *Candida* culture was obtained <3 days after acute care hospital admission or in the outpatient setting in a patient with no recent health care exposures, “health care onset” if the culture was obtained ≥3 days after hospital admission, or “health care–associated community onset” if the culture was obtained <3 days after hospital admission in a patient with a recent health care exposure. Recent health care exposure was defined as an overnight stay in a nursing home, hospitalization at an acute care hospital (including intensive care unit [ICU] stays and excluding emergency department visits or outpatient procedures) during the 90 days before the index date, surgery during the 90 days before the index date, or hemodialysis receipt during the 30 days before the index date.

### Laboratory Methods

*Candida* isolates associated with candidemia cases were obtained and sent by EIP sites to CDC to confirm species identification and perform antifungal susceptibility testing (AFST). CDC laboratory results were not used for clinical care. CDC’s fungal reference laboratory confirmed species identification using matrix-assisted laser desorption/ionization time-of-flight mass spectrometry (MALDI-TOF) or DNA sequencing. AFST was performed using prepared microdilution plates for fluconazole, voriconazole, anidulafungin, caspofungin, and micafungin according to guidelines in the Clinical and Laboratory Standards Institute (CLSI) M27 standard (*21*). Amphotericin B susceptibility was performed using gradient diffusion. Minimum inhibitory concentrations were reported and resistance interpreted using species-specific breakpoints outlined in the CLSI M60 standard (*22*). Echinocandin resistance was defined as resistance to anidulafungin, caspofungin, or micafungin. Multidrug resistance was defined as resistance to fluconazole and any echinocandin. For this report, CDC testing results were used when available. If an isolate was unavailable or was not viable upon arrival at CDC, the species identification from the clinical laboratory was used. However, for AFST, only CDC data were used because of variability in AFST methods at the local clinical laboratories serving the surveillance areas.

### Analysis

Case counts and frequencies were tabulated, and descriptive statistics were examined by year, stratifying by demographic characteristics, underlying medical conditions, candidemia risk factors, health care use, treatment, outcomes, species distribution, and AFST. Annual incidence rates per 100,000 population were calculated using yearly U.S. Census Bureau population estimates (https://data.census.gov) and were stratified by age group, sex, U.S. census region, and surveillance site. Cases missing the index date (<1%) were excluded from this analysis. This activity was reviewed by CDC, deemed not research, and was conducted consistent with applicable Federal law and CDC policy.* 

## Results

### Demographic Characteristics and Incidence

During 2017–2021, a total of 7,381 candidemia cases from 6,235 patients were identified. Among patients with multiple cases (n = 1,146), 377 (32.9%) patients had two cases, 145 (12.7%) had three cases, 108 (9.4%) had four cases, and 516 (45.0%) had ≥5 cases. Demographic characteristics of patients with candidemia were generally similar across years, other than higher proportions in 2021 compared with 2017 of patients aged ≥65 years (45.6% versus 40.2%) and those who were Black or African American (Black) (29.8% versus 24.8%) (Table 1). Proportions of White patients with candidemia were slightly lower (44.8% versus 53.1%) whereas proportions of Asian (3.0% versus 1.6%) and Hispanic or Latino patients (4.3% versus 2.2%) were higher. Overall, 42.2% of patients were aged ≥65 years, 35.5% were aged 45–64 years, 18.7% were aged 19–44 years, 1.8% were aged 1–18 years, and 1.7% were aged <1 year. Overall, 56.3% of patients were male; 49.1% of patients were White, 27.6% were Black, 3.7% were Hispanic, 2.7% were Asian, 1.4% were another race, and 15.4% were of unknown race and ethnicity. (Persons of Hispanic or Latino origin might be of any race but are categorized as Hispanic; all racial groups are non-Hispanic.) The Georgia EIP site had the most cases (n = 1,828 [24.8%]), followed by the sites in Maryland (n = 963 [13.0%]), Connecticut (n = 825 [11.2%]), Colorado (n = 799 [10.8%]), Tennessee (n = 796 [10.8%]), Minnesota (n = 786 [10.6%]), California (n = 484 [6.6%]), Oregon (n = 360 [4.9%]), New York (n = 331 [4.5%]), and New Mexico (n = 209 [2.8%]).

**TABLE 1:** Demographic characteristics of patients with candidemia and number of cases from each surveillance site, by year — 10 sites, United States, 2017–2021*^,†^

Patient characteristic/Site	2017 (n = 1,168)	2018 (n = 1,185)	2019 (n = 1,135)	2020 (n = 1,382)	2021 (n = 1,365)	Total (N = 6,235)
	No. (%)	No. (%)	No. (%)	No. (%)	No. (%)	No. (%)
**Median age, yrs (IQR)**	60 (45–70)	60 (45–71)	60 (45–72)	62 (49–72)	63 (49–74)	**61 (47–72)**
**Age group,^†^ yrs**						
<1	18 (1.5)	26 (2.2)	22 (1.9)	23 (1.7)	17 (1.2)	**106 (1.7)**
1–18	13 (1.1)	30 (2.5)	21 (1.9)	22 (1.6)	27 (2.0)	**113 (1.8)**
19–44	256 (21.9)	237 (20.0)	234 (20.6)	221 (16.0)	217 (15.9)	**1,165 (18.7)**
45–64	412 (35.3)	418 (35.3)	395 (34.8)	512 (37.1)	476 (34.9)	**2,213 (35.5)**
≥65	469 (40.2)	474 (40.0)	463 (40.8)	604 (43.7)	622 (45.6)	**2,632 (42.2)**
Unknown	0 (—)	0 (—)	0 (—)	0 (—)	6 (0.4)	**6 (0.1)**
**Sex**						
Female	521 (44.6)	529 (44.6)	485 (42.7)	586 (42.4)	605 (44.3)	**2,726 (43.7)**
Male	647 (55.4)	656 (55.4)	650(57.3)	796 (57.6)	760 (55.7)	**3,509 (56.3)**
**Race and ethnicity^§^**						
Asian	19 (1.6)	28 (2.4)	38 (3.3)	45 (3.3)	41 (3.0)	**171 (2.7)**
Black	290 (24.8)	325 (27.4)	305 (26.9)	396 (28.7)	407 (29.8)	**1,723 (27.6)**
White	620 (53.1)	596 (50.3)	573 (50.5)	619 (44.8)	654 (47.9)	**3,062 (49.1)**
Other races^¶^	16 (1.4)	14 (1.2)	15 (1.3)	27 (2.0)	18 (1.3)	**90 (1.4)**
Hispanic or Latino	26 (2.2)	36 (3.0)	38 (3.3)	70 (5.1)	59 (4.3)	**229 (3.7)**
Unknown	197 (16.9)	186 (15.7)	166 (14.6)	225 (16.3)	186 (13.6)	**960 (15.4)**
**Surveillance site**	**(n = 1,226)**	**(n = 1,275)**	**(n = 1,473)**	**(n = 1,699)**	**(n = 1,708)**	**(N = 7,381)**
California	83 (6.8)	107 (8.4)	97 (6.6)	104 (6.1)	93 (5.4)	**484 (6.6)**
Colorado	115 (9.4)	134 (10.5)	172 (11.7)	200 (11.8)	178 (10.4)	**799 (10.8)**
Connecticut**	NA	NA	275 (18.7)	258 (15.2)	292 (17.1)	**825 (11.2)**
Georgia	311 (25.4)	360 (28.2)	312 (21.2)	426 (25.1)	419 (24.5)	**1,828 (24.8)**
Maryland	204 (16.6)	199 (15.6)	169 (11.5)	184 (10.8)	207 (12.1)	**963 (13.0)**
Minnesota	143 (11.7)	139 (10.9)	160 (10.9)	186 (10.9)	158 (9.3)	**786 (10.6)**
New Mexico	39 (3.2)	42 (3.3)	40 (2.7)	43 (2.5)	45 (2.6)	**209 (2.8)**
New York	70 (5.7)	50 (3.9)	59 (4.0)	79 (4.6)	73 (4.3)	**331 (4.5)**
Oregon	89 (7.3)	68 (5.3)	55 (3.7)	60 (3.5)	88 (5.2)	**360 (4.9)**
Tennessee	172 (14.0)	176 (13.8)	134 (9.1)	159 (9.4)	155 (9.1)	**796 (10.8)**

**Abbreviation:** NA = not applicable.

* During 2017–2021, a total of 7,381 candidemia cases from 6,235 patients were identified.

^†^ Data shown as no. (%) column unless otherwise indicated.

^§^ Persons of Hispanic or Latino (Hispanic) origin might be of any race but are categorized as Hispanic; all racial groups are non-Hispanic.

^¶^ Includes American Indian, Native Hawaiian, and multiracial.

** Connecticut is the only statewide surveillance site and started reporting in 2019.

**FIGURE 1:**
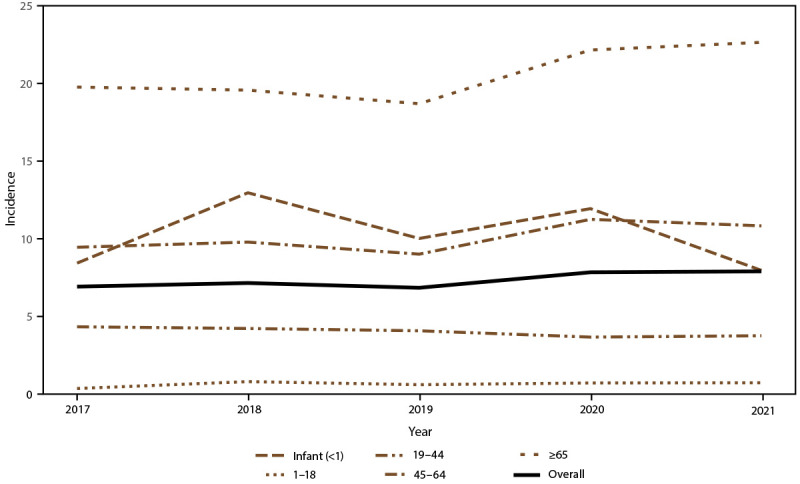
Annual candidemia incidence,* overall and by age group — 10 sites,^†^ United States, 2017–2021 * Per 100,000 population, calculated from the U.S. Census Bureau population and housing unit estimates for the corresponding years (https://data.census.gov). ^†^ California, Colorado, Connecticut, Georgia, Maryland, Minnesota, New Mexico, New York, Oregon, and Tennessee. Connecticut is the only statewide surveillance site and started reporting in 2019.

**FIGURE 2:**
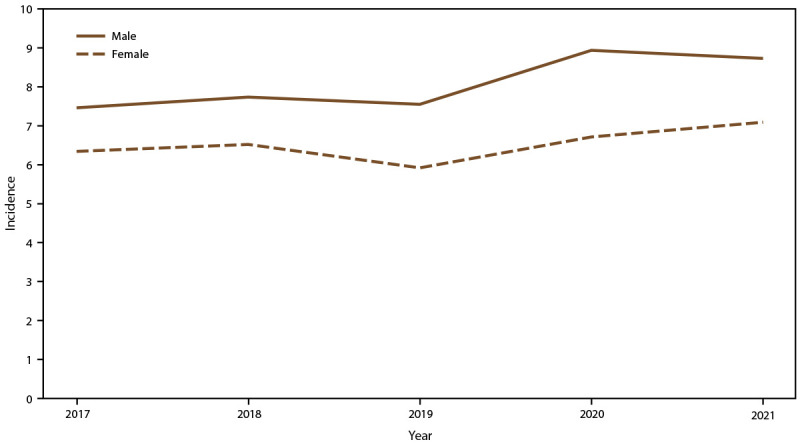
Annual candidemia incidence,* by sex — 10 sites,^†^ United States, 2017–2021 * Per 100,000 population, calculated from the U.S. Census Bureau population and housing unit estimates for the corresponding years (https://data.census.gov). ^†^ California, Colorado, Connecticut, Georgia, Maryland, Minnesota, New Mexico, New York, Oregon, and Tennessee. Connecticut is the only statewide surveillance site and started reporting in 2019.

**FIGURE 3:**
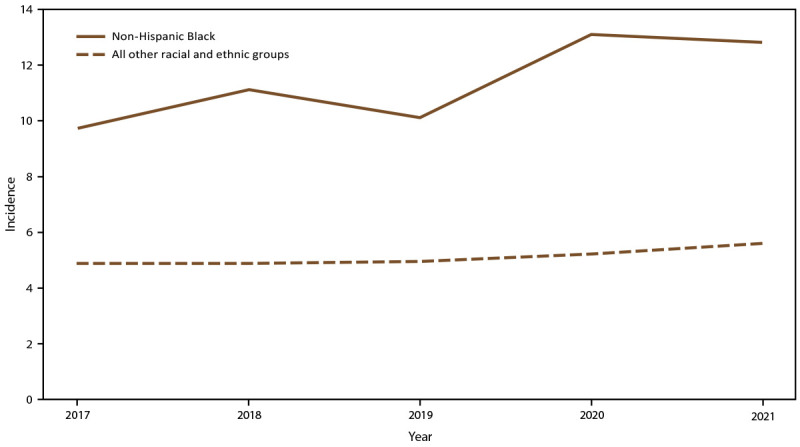
Annual candidemia incidence,* by race and ethnicity — 10 sites,^†^ United States, 2017–2021 * Per 100,000 population, calculated from the U.S. Census Bureau population and housing unit estimates for the corresponding years (https://data.census.gov). ^†^ California, Colorado, Connecticut, Georgia, Maryland, Minnesota, New Mexico, New York, Oregon, and Tennessee. Connecticut is the only statewide surveillance site and started reporting in 2019.

**FIGURE 4:**
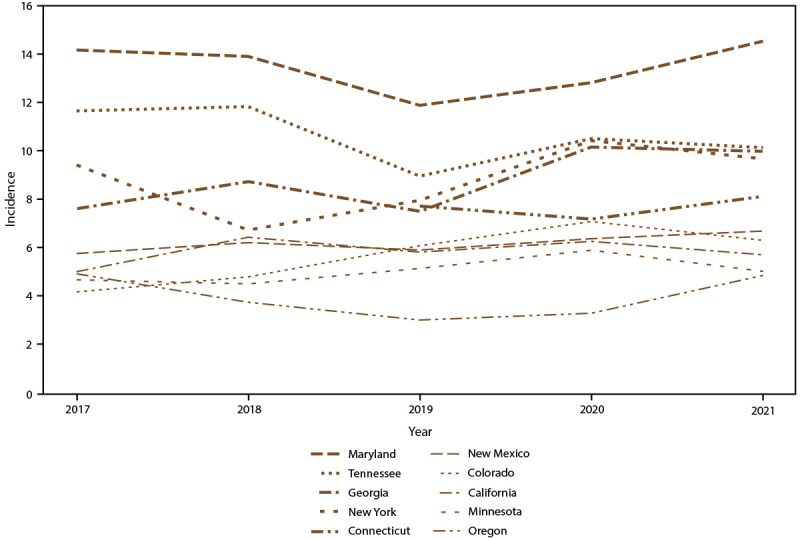
Annual candidemia incidence,* by site — 10 sites,^†^ United States, 2017–2021 * Per 100,000 population, calculated from the U.S. Census Bureau population and housing unit estimates for the corresponding years (https://data.census.gov). ^†^ Connecticut is the only statewide surveillance site and started reporting in 2019.

Overall candidemia incidence during 2017–2021 was 7.4 cases per 100,000 population. Incidence was generally stable across years, with the lowest incidence in 2019 (6.8) and the highest in 2021 (7.9) (Figure 1). Across age groups, sexes, racial and ethnic groups, and surveillance sites, incidence was generally stable or increased slightly from 2017 to 2021. In 2021, candidemia incidence was highest in patients aged ≥65 years (22.7) and infants (aged <1 year) (8.0) (Figure 1); higher in males (8.7) compared with females (7.0) (Figure 2); higher in Black patients (12.8) compared with non-Black patients (5.6) (Figure 3); and highest in Maryland (14.5), followed by Tennessee (10.1) and Georgia (10.0), with incidence lowest in Oregon (4.8) (Figure 4).

### Clinical Characteristics, Underlying Conditions, and Risk Factors for Candidemia

The percentage of cases classified as health care onset was higher in 2021 (58.0%) versus 2017 (52.2%) (Table 2). The median time from admission to index date was 4 (IQR = 0–15) days. The percentage of cases involving an ICU stay was also higher in 2021 (44.9%) versus 2017 (38.3%).

**TABLE 2:** Classification of candidemia cases, days from admission to blood culture obtained, and recent health care stays, by year — 10 sites,* United States, 2017–2021^†^

Characteristic	2017 (n = 1,226)	2018 (n = 1,275)	2019 (n = 1,473)	2020 (n = 1,699)	2021 (n = 1,708)	Total (N = 7,381)
	No. (%)	No. (%)	No. (%)	No. (%)	No. (%)	No. (%)
**Case classification^§^**						
Community onset	162 (13.2)	179 (14.0)	190 (12.9)	215 (12.7)	220 (12.9)	**966 (13.1)**
Health care–associated community onset	424 (34.6)	459 (36.0)	548 (37.2)	510 (30.0)	497 (29.1)	**2,438 (33.0)**
Health care onset	640 (52.2)	637 (50.0)	735 (49.9)	974 (57.3)	991 (58.0)	**3,977 (53.9)**
Median days from admission to index culture (IQR)	3 (0–15)	3 (0–13)	3 (0–13)	5 (0–16)	6 (0–17)	**4 (0–15)**
ICU stay during the 14 days before index date	469 (38.3)	516 (40.5)	579 (39.3)	773 (45.5)	767 (44.9)	**3,104 (42.1)**
**Overnight health care stay during the 90 days before date of collection for initial blood culture positive for *Candida***						
Hospitalization (acute care hospital)	681 (55.5)	671 (52.6)	797 (54.1)	771 (45.4)	804 (47.1)	**3,724 (50.5)**
Overnight long-term care facility stay (n = 6,155)	NA	220 (17.3)	280 (19.0)	262 (15.4)	269 (15.7)	**1,031 (16.8)**
Overnight long-term acute care hospital stay (n = 6,155)	NA	46 (3.6)	51 (3.5)	43 (2.5)	60 (3.5)	**200 (3.2)**

**Abbreviation:** ICU = intensive care unit; NA = not applicable.

* California, Colorado, Connecticut, Georgia, Maryland, Minnesota, New Mexico, New York, Oregon, and Tennessee. Connecticut is the only statewide surveillance site and started reporting in 2019.

^†^ Data shown as no. (%) unless otherwise indicated. Index date was the specimen collection date for the initial positive *Candida* blood culture.

^§^ Cases were classified as “community onset” if the initial positive *Candida* culture was obtained <3 days after acute care hospital admission or in the outpatient setting in a patient with no recent health care exposures, “health care onset” if the culture was obtained ≥3 days after hospital admission, or “health care–associated community onset” if the culture was obtained <3 days after hospital admission in a patient with a recent health care exposure. Recent health care exposure was defined as overnight stay in a nursing home, hospitalization at an acute care hospital (including intensive care unit [ICU] stays and excluding emergency department visits or outpatient procedures) during the 90 days before the index date, surgery during the 90 days before the index date, or hemodialysis receipt during the 30 days before the index date.

The most common underlying condition was diabetes (36.2%), with the frequency increasing from 32.1% (2017) to 38.0% (2021) (Table 3). Frequencies of other underlying conditions were generally stable over time. Overall, 25.6% of patients had chronic kidney disease, 24.3% had malignancy, 22.1% had chronic lung disease, 16.6% had liver disease (with the percentage higher in 2017 [18.2%] compared with 2021 [15.6%] and the percentage with hepatitis C infection higher in 2017 [11.4%] compared with 2021 [7.7%]), 28.4% reported smoking, 10.4% had a history of alcohol use disorder, 9.0% had injection drug use (with the percentage higher in 2017 [10.7%] compared with 2021 [7.1%]), 29.2% had recent surgery, 63.7% had a central venous catheter, 80.7% received recent systemic antibiotics, and 19.6% received total parenteral nutrition. From 2020 to 2021, the percentage of cases with a positive SARS-CoV-2 test during the 90 days surrounding the index date increased from 10.4% to 17.7%.

**TABLE 3:** Underlying medical conditions, substance use, and other risk factors associated with candidemia cases, by year — 10 sites,* United States, 2017–2021^†^

Risk factor	2017 (n = 1,226)	2018 (n = 1,275)	2019 (n = 1,473)	2020 (n = 1,699)	2021 (n = 1,708)	Total (N = 7,381)
	No. (%)	No. (%)	No. (%)	No. (%)	No. (%)	No. (%)
**Underlying medical condition**						
Diabetes	394 (32.1)	453 (35.5)	536 (36.4)	638 (37.6)	649 (38.0)	**2,670 (36.2)**
Chronic kidney disease	325 (26.5)	314 (24.6)	389 (26.4)	428 (25.2)	436 (25.5)	**1,892 (25.6)**
Chronic lung disease	254 (20.7)	297 (23.3)	328 (22.3)	367 (21.6)	385 (22.5)	**1,631 (22.1)**
Malignancy (n = 6,155)	NA	306 (24.0)	372 (25.3)	405 (23.8)	411 (24.1)	**1,494 (24.3)**
Liver disease	223 (18.2)	199 (15.6)	266 (18.1)	268 (15.8)	266 (15.6)	**1,222 (16.6)**
Gastrointestinal disease (n = 6,155)	NA	147 (11.5)	157 (10.7)	165 (9.7)	187 (11.0)	**656 (10.7)**
Dialysis (n = 6,085)	NA	113 (8.9)	139 (9.5)	144 (8.5)	170 (10.0)	**566 (9.3)**
Hepatitis C infection	140 (11.4)	115 (9.0)	154 (10.5)	140 (8.2)	132 (7.7)	**681 (9.2)**
Transplant (hematopoietic stem cell or solid organ)	28 (2.3)	41 (3.2)	54 (3.7)	67 (3.9)	34 (2.0)	**224 (3.0)**
HIV infection	29 (2.4)	29 (2.3)	26 (1.8)	32 (1.9)	24 (1.4)	**140 (1.9)**
Neutropenia during the 2 days before index date (n = 6,073)	47 (5.2)	53 (5.0)	66 (5.4)	75 (5.3)	78 (5.3)	**319 (5.3)**
**Substance use**						
Smoking^§^	289 (23.6)	330 (25.9)	481 (32.7)	500 (29.4)	498 (29.2)	**2,098 (28.4)**
History of alcohol use disorder	85 (6.9)	122 (9.6)	155 (10.5)	182 (10.7)	225 (13.2)	**769 (10.4)**
Injection drug use during 12 months before index date	131 (10.7)	120 (9.4)	188 (12.8)	106 (6.2)	121 (7.1)	**666 (9.0)**
**Other risk factor**						
Any surgery during the 90 days before index date	411 (33.5)	418 (32.8)	431 (29.3)	425 (25.0)	472 (27.6)	**2,157 (29.2)**
Abdominal surgery	222 (18.1)	237 (18.6)	255 (17.3)	253 (14.9)	258 (15.1)	**1,225 (16.6)**
Central venous catheter	833 (67.9)	872 (68.4)	877 (59.5)	1,046 (61.6)	1,075 (62.9)	**4,703 (63.7)**
Central venous catheter removed or changed within 7 days of index date (n = 3,855)	536 (76.8)	499 (68.8)	562 (75.7)	629 (75.0)	634 (74.5)	**2,860 (74.2)**
Systemic antibiotics during 14 days before index date	1,014 (82.7)	1,054 (82.7)	1,131 (76.8)	1,338 (78.8)	1,419 (83.1)	**5,956 (80.7)**
Total parenteral nutrition during the 14 days before index date	269 (21.9)	285 (22.4)	274 (18.6)	295 (17.4)	327 (19.1)	**1,450 (19.6)**
SARS-CoV-2 positive test during the 90 days before or after index date (n = 3,047)	NA	NA	NA	176 (10.4)	302 (17.7)	**478 (15.7)**

**Abbreviation:** NA = not applicable.

* California, Colorado, Connecticut, Georgia, Maryland, Minnesota, New Mexico, New York, Oregon, and Tennessee. Connecticut is the only statewide surveillance site and started reporting in 2019. Data are for 6,235 persons, and variables are not mutually exclusive.

^†^ Data shown as no. (%) column. Index date was the specimen collection date for the initial positive *Candida* blood culture.

^§^ During 2019–2020, included tobacco, e-nicotine delivery system, and marijuana.

### Antifungal Treatment, Health Care Use, Clinical Course, and Outcomes

The most common antifungals received were echinocandins (49.8%), followed by azoles (47.7%) and amphotericin B (4.4%) (Table 4). The percentage of cases involving no antifungal treatment increased from 18.0% in 2017 to 35.4% in 2020 and decreased to 30.1% in 2021. The most common reason for not receiving antifungal treatment was that the patient died before the culture result was available to clinicians (52.7% of those not treated). Candidemia-associated complications included endocarditis (4.0%), followed by abscess (2.4%), osteomyelitis (1.4%), septic embolus (1.0%), and endophthalmitis or chorioretinitis (0.9%). Overall, 24.8% of cases had non-*Candida* organisms isolated from blood cultures during the 6 days before the index date; this percentage increased from 17.8% in 2017 to 28.7% in 2021. The most common non-*Candida* pathogen was *Enterococcus faecium* (8.6%), followed by *Staphylococcus aureus* (8.3%) and *Escherichia coli* (7.9%). *Staphylococcus epidermidis*, a common blood culture contaminant, was cultured in 11.7%.

**TABLE 4:** Antifungal treatment, other body sites with *Candida* detection, additional organisms isolated from blood culture, health care use, and clinical outcome for candidemia cases, by year — 10 sites,* United States, 2017–2021^†^

Characteristic	2017 (n = 1,226)	2018 (n = 1,275)	2019 (n = 1,473)	2020 (n = 1,699)	2021 (n = 1,708)	Total (N = 7,381)
	No. (%)	No. (%)	No. (%)	No. (%)	No. (%)	No. (%)
**Antifungal treatment (not mutually exclusive)**						
Echinocandins	780 (63.6)	836 (65.6)	632 (42.9)	663 (39.0)	764 (44.7)	**3,675 (49.8)**
Azoles	641 (52.3)	625 (49.0)	754 (51.2)	723 (42.6)	781 (45.7)	**3,524 (47.7)**
Amphotericin B	47 (3.8)	73 (5.7)	71 (4.8)	70 (4.1)	62 (3.6)	**323 (4.4)**
Other	13 (1.1)	18 (1.4)	9 (0.6)	10 (0.6)	14 (0.8)	**64 (0.9)**
No treatment**^§^**	221 (18.0)	265 (20.8)	406 (27.6)	601 (35.4)	514 (30.1)	**2,007 (27.2)**
**Other site of *Candida* infection^¶^ (n = 4,880)**						
Abscess	NA	NA	16 (1.1)	70 (4.1)	32 (1.9)	**118 (2.4)**
Eye (endophthalmitis or chorioretinitis)	NA	NA	14 (1.0)	14 (0.8)	18 (1.1)	**46 (0.9)**
Endocarditis	NA	NA	71 (4.8)	60 (3.5)	66 (3.9)	**197 (4.0)**
Septic embolus	NA	NA	8 (0.5)	26 (1.5)	14 (0.8)	**48 (1.0)**
Osteomyelitis	NA	NA	27 (1.8)	26 (1.5)	15 (0.9)	**68 (1.4)**
**Non-*Candida* sp. organism isolated 6 days before index date**	218 (17.8)	239 (18.7)	404 (27.4)	480 (28.3)	491 (28.7)	**1,832 (24.8)**
*Staphylococcus epidermidis*	25 (11.5)	22 (9.2)	42 (10.4)	62 (12.9)	63 (12.8)	**214 (11.7)**
*Enterococcus faecium*	23 (10.6)	16 (6.7)	35 (8.7)	34 (7.1)	50 (10.2)	**158 (8.6)**
*Staphylococcus aureus*	14 (6.4)	19 (7.9)	36 (8.9)	38 (7.9)	45 (9.2)	**152 (8.3)**
*Escherichia coli*	7 (3.2)	15 (6.3)	39 (9.7)	42 (8.8)	41 (8.4)	**144 (7.9)**
**Health care use**						
Current hospitalization for candidemia	1,184 (96.6)	1,209 (94.8)	1,415 (96.1)	1,632 (96.1)	1,649 (96.5)	**7,089 (96.0)**
ICU stay during the 13 days after candidemia diagnosis	637 (52.0)	679 (53.3)	811 (55.1)	996 (58.6)	944 (55.3)	**4,067 (55.1)**
Total length of hospital stay, days (IQR)	15 (7–34)	15 (7–31)	15 (7–31)	16 (8–34)	18 (9–36)	**16 (7–33)**
Length of hospital stay after candidemia diagnosis, days (IQR)	9 (5–19)	9 (4–19)	9 (5–18)	9 (4–18)	10 (4–20)	**9 (5–19)**
**Clinical outcome**						
In-hospital death	328 (26.8)	406 (31.8)	446 (30.3)	612 (36.0)	616 (36.1)	**2,408 (32.6)**
Death within 48 hours after first positive *Candida* culture	96 (7.8)	143 (11.2)	118 (8.0)	215 (12.7)	199 (11.7)	**771 (10.4)**
Median days from positive *Candida* culture to death (IQR)	6 (2–16)	5 (2–14)	6 (2–13)	5 (2–12)	5 (2–13)	**5 (2–13)**

**Abbreviation:** ICU = intensive care unit; NA = not applicable.

* California, Colorado, Connecticut, Georgia, Maryland, Minnesota, New Mexico, New York, Oregon, and Tennessee. Connecticut is the only statewide surveillance site and started reporting in 2019.

^†^ Data shown as no. (%) column unless otherwise indicated.

^§^ Reason for not receiving antifungal treatment was available for 1,057 (52.7%) cases and included the following: the patient died before culture results were available (51.6%), the patient was on comfort care measures only (19.4%), the patient was discharged before culture results were available (19.0%), the clinical team considered the culture result to not be clinically significant or to represent contamination (4.4%), the patient refused treatment (2.6%), and other reasons documented in the medical chart (3.0%).

^¶^ Information on abscess and osteomyelitis were missing for 207 patients in 2021.

Overall, 55.1% of cases involved an ICU stay on the day of or within 13 days after the index date; median length of hospitalization was 16 (IQR = 7–33) days, and 32.6% of cases involved in-hospital death. The percentage of cases involving death increased from 26.8% in 2019 to 36.1% in 2021.

### Species Distribution and AFST

**FIGURE 5:**
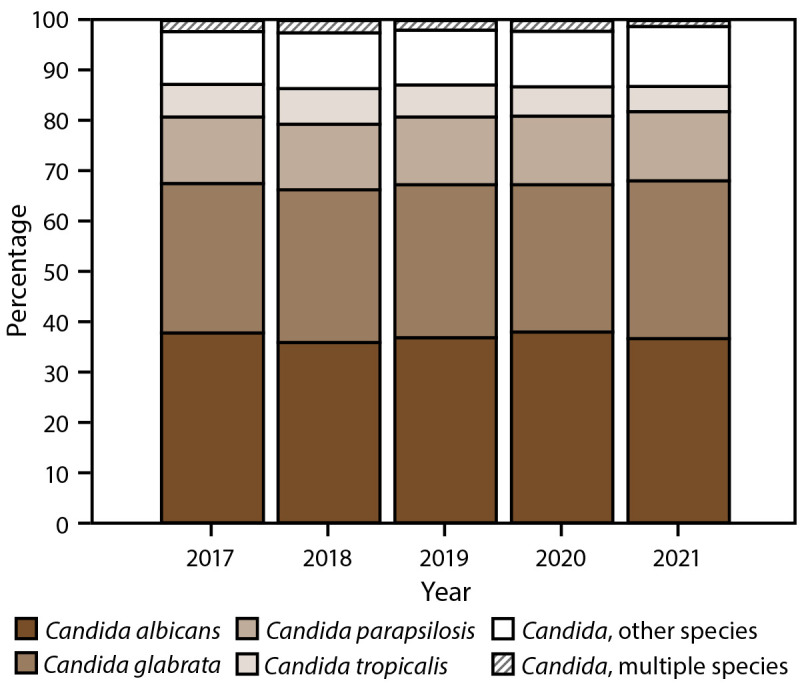
*Candida* species* distribution, by year — 10 sites,^†^ United States, 2017–2021 * The category “*Candida*, other species” includes *C. bracarensis, C. cacao, C. catenulata, C. dubliniensis, C. duobushaemulonii, C. famata, C. fermentati, C. glaebosa, C. guilliermondii, C. guilliermondii* var. membrane*, C. intermedia, C. kefyr, C. krusei, C. lipolytica, C. lusitaniae, C. magnoliae, C. metapsilosis, C. nivariensis, C. orthopsilosis, C. pararugosa, C. pelliculosa, C. quercitrusa, C. rugosa, C. spherica, C. sojae, C. sorbosa, C. sorbosivorans, C. utilis, Candida* germ tube negative, and unknown. ^†^ California, Colorado, Connecticut, Georgia, Maryland, Minnesota, New Mexico, New York, Oregon, and Tennessee. Connecticut is the only statewide surveillance site and started reporting in 2019.

The overall *Candida* spp. distribution remained stable across years. The most common species was *C. albicans* (37.1%), followed by *C. glabrata* (30.4%), *C*. *parapsilosis* (13.5%), and *C. tropicalis* (6.1%); 11.1% of cases involved another *Candida* sp., and 1.9% involved multiple species (Figure 5). *C. auris* was identified in 0.4% of cases with no difference in prevalence across years or sites. *C. albicans* was the most frequent species at all but two surveillance sites; *C. glabrata* was the most frequent species in California (38.4%) and Maryland (32.9%). Species distributions remained similar across years for each site except for New Mexico, where the percentage of *C. glabrata* isolates increased from 15.4% (2017) to 48.9% (2021).

Among 6,576 isolates tested, 5.6% were fluconazole resistant, <1% were voriconazole resistant, <1% were echinocandin resistant, and <1% were multidrug resistant (Table 5). Fluconazole resistance was most frequent for *C. parapsilosis* isolates (7.5%), followed by *C. glabrata* isolates (4.9%) and *C. tropicalis* isolates (4.0%). The percentage of antifungal-resistant isolates was stable across years.

**TABLE 5:** Drug resistance in *Candida* isolates,* by species and year — 10 sites,^†^ United States, 2017–2021

Species/Drug	2017	2018	2019	2020	2021	Total
	No. (%)	No. (%)	No. (%)	No. (%)	No. (%)	No. (%)
***Candida albicans***	**n = 463**	**n = 458**	**n = 543**	**n = 645**	**n = 626**	**N = 2,735**
Amphotericin B	0 (—)	0 (—)	0 (—)	0 (—)	0 (—)	**0 (—)**
Fluconazole	2 (0.4)	1 (0.2)	2 (0.4)	4 (0.6)	2 (0.3)	**11 (0.4)**
Voriconazole	1 (0.2)	1 (0.2)	0 (—)	0 (—)	0 (—)	**2 (0.1)**
Echinocandins^§^	1 (0.2)	1 (0.2)	1 (0.2)	0 (—)	0 (—)	**3 (0.1)**
Multiple drugs^¶^	0 (—)	0 (—)	0 (—)	0 (—)	0 (—)	**0 (—)**
***Candida glabrata***	**n = 365**	**n = 388**	**n = 449**	**n = 500**	**n = 538**	**N = 2,240**
Amphotericin B	0 (—)	0 (—)	0 (—)	0 (—)	0 (—)	**0 (—)**
Fluconazole	21 (5.8)	21 (5.4)	29 (6.5)	19 (3.8)	19 (3.5)	**109 (4.9)**
Voriconazole**	NA	NA	NA	NA	NA	**NA**
Echinocandins^§^	11 (3.0)	6 (1.5)	8 (1.8)	8 (1.6)	12 (2.2)	**45 (2.0)**
Multiple drugs^¶^	1 (0.3)	1 (0.3)	4 (0.9)	2 (0.4)	2 (0.4)	**10 (0.4)**
***Candida krusei***	**n = 21**	**n = 29**	**n = 29**	**n = 38**	**n = 41**	**N = 158**
Amphotericin B	0 (—)	0 (—)	0 (—)	0 (—)	0 (—)	**0 (—)**
Fluconazole^††^	NA	NA	NA	NA	NA	**NA**
Voriconazole	1 (4.8)	0 (—)	0 (—)	2 (5.3)	1 (2.4)	**4 (2.5)**
Echinocandins^§^	0 (—)	0 (—)	0 (—)	0 (—)	0 (—)	**0 (—)**
Multiple drugs^¶^	0 (—)	0 (—)	0 (—)	0 (—)	0 (—)	**0 (—)**
***Candida parapsilosis***	**n = 162**	**n = 167**	**n = 199**	**n = 232**	**n = 235**	**N = 995**
Amphotericin B	0 (—)	0 (—)	0 (—)	0 (—)	0 (—)	**0 (—)**
Fluconazole	12 (7.4)	10 (6.0)	11 (5.5)	19 (8.2)	23 (9.8)	**75 (7.5)**
Voriconazole	1 (0.6)	4 (2.4)	6 (3.0)	9 (3.9)	9 (3.8)	**29 (2.9)**
Echinocandins^§^	0 (—)	0 (—)	0 (—)	1 (0.4)	7 (3.0)	**8 (0.8)**
Multiple drugs^¶^	0 (—)	0 (—)	0 (—)	1 (0.4)	0 (—)	**1 (0.1)**
***Candida tropicalis***	**n = 80**	**n = 91**	**n = 94**	**n = 98**	**n = 85**	**N = 448**
Amphotericin B	0 (—)	0 (—)	0 (—)	0 (—)	0 (—)	**0 (—)**
Fluconazole	4 (5.0)	2 (2.2)	7 (7.4)	3 (3.1)	2 (2.4)	**18 (4.0)**
Voriconazole	2 (2.5)	2 (2.2)	5 (5.3)	1 (1.0)	2 (2.4)	**12 (2.7)**
Echinocandins^§^	0 (—)	0 (—)	2 (2.1)	0 (—)	0 (—)	**2 (0.4)**
Multiple drugs^¶^	0 (—)	0 (—)	0 (—)	0 (—)	0 (—)	**0 (—)**

**Abbreviation:** NA = not applicable.

* Only includes data on isolates sent to CDC for the top five *Candida* spp. *Candida auris* is not included in the table because specific *C. auris* susceptibility breakpoints do not exist. Breakpoints were defined by CDC on the basis of those established for closely related *Candida* sp. and on expert opinion (https://www.cdc.gov/candida-auris/hcp/laboratories/antifungal-susceptibility-testing.html). Among the two *C. auris* isolates identified in the surveillance system, one was considered fluconazole resistant on the basis of tentative breakpoints.

^†^ California, Colorado, Connecticut, Georgia, Maryland, Minnesota, New Mexico, New York, Oregon, and Tennessee. Connecticut is the only statewide surveillance site and started reporting in 2019.

^§^ Defined as resistance to any of the echinocandins (anidulafungin, caspofungin, and micafungin).

^¶^ Defined as resistance to any echinocandin and fluconazole.

** No breakpoints.

^††^ *Candida krusei* has intrinsic resistance to fluconazole.

## Discussion

This report summarizes the incidence, underlying conditions, health care exposures, treatment, species distribution, antifungal resistance, and outcomes associated with approximately 7,400 candidemia cases at 10 CDC EIP surveillance sites during 2017–2021. The overall candidemia incidence across sites and years was 7.4 per 100,000 population, which is slightly lower compared with findings from surveillance data collected during 2012–2016 (8.7) (*3*). During 2021, the all-cause mortality rate associated with candidemia was higher compared with earlier years (36.1% versus 26.8% in 2017 and 25% during 2012–2016), and cases during 2020–2021 also more frequently involved previous ICU stays and onset in the health care setting. These findings might reflect the effects of the COVID-19 pandemic during this period because patients with COVID-19–associated infections were more likely to be critically ill and have worse outcomes compared with those without COVID-19 (*23*).

Differences in candidemia incidence by age, sex, racial and ethnic group, and geographic region were consistent with previous surveillance data (*3*). Candidemia incidence remained the highest in persons aged ≥65 years (22.7 per 100,000 population during 2017–2021), and the percentage of older adults represented in surveillance data increased during 2020–2021. This finding likely reflects the occurrence of COVID-19–associated candidemia cases during this time frame because severe COVID-19 more often affects older adults (*23*). More men than women had candidemia cases, a finding consistent across previous studies (*3*,*24*). Reasons for this finding are unclear but might relate to sex-dependent host immune responses, hormonal factors, or differences in behavioral characteristics that might affect risk (*24*). This surveillance effort also aligns with previous findings regarding the disproportionate incidence of candidemia in Black patients (*3*). The percentage of Black patients affected by candidemia increased during the study period, likely mirroring the disproportionate effect of the COVID-19 pandemic on Black patients (*3*,*17*,*25*). Incidence of candidemia varied widely among sites (approximately three times higher in Maryland versus Oregon), reflecting the importance of understanding local epidemiology because candidemia incidence and species distribution might differ regionally. The frequencies of candidemia risk factors were similar to previously published EIP surveillance findings and were generally similar throughout the surveillance period. Diabetes, malignancies, renal disease, recent surgery, and other established risk factors continued to be frequent among patients with candidemia (*3*), and slight decreases occurred in the percentages of patients with chronic liver disease, hepatitis C infection, and injection drug use.

Overall, *C. albicans* remained the most common *Candida* species (37.1%), similar to previous findings (39%) (*3*). However, *C. glabrata* was the predominant species in California (38.4%) and Maryland (32.9%), and the percentage of *C. glabrata* isolates identified in New Mexico approximately tripled during the surveillance period. Although *C. auris* remains a major public health threat in the United States, it was infrequently detected in this surveillance system (<1% of cases), perhaps because much of the transmission occurring was outside the surveillance catchment area. On the basis of the increasing frequency of invasive candidiasis caused by non-*C. albicans* species, concern about fluconazole resistance, and evidence of echinocandins being more effective than fluconazole, the Infectious Diseases Society of America guidelines recommend echinocandins as the preferred initial therapy for most patients with candidemia (*1*). However, similar to surveillance findings during 2012–2016 (*3*), only 49.8% of candidemia cases involved echinocandin treatment, likely because certain patients died before treating health care providers knew about the candidemia diagnosis or because of low awareness of echinocandins as the preferred first-line therapy for most candidemia patients (*26*). This finding underscores the continued importance of candidemia surveillance to monitor treatment practices because approximately 5% of *C*. *glabrata* isolates were resistant to fluconazole.

## Limitations

The findings in this report are subject to at least five limitations. First, data were reported from only 10 geographic areas, limiting representativeness. Second, changes in data collection over the years limited the availability, and therefore interpretation, of certain data across all years. Third, although data were extracted from medical charts by trained surveillance officers, personnel might interpret data provided in medical charts differently, potentially leading to inconsistencies in chart abstraction. Fourth, certain data elements are not systematically or clearly documented in medical charts, likely resulting in an underestimation of risk factors. Finally, whereas blood cultures are the gold standard for diagnosing invasive candidiasis, their sensitivity is low, meaning certain cases might not have been detected and therefore not included as part of this candidemia surveillance.

## Conclusion

Candidemia remains an important health care–associated infection that continues to disproportionately affect older adults, males, and Black patients. The overall candidemia incidence and percentage distribution for most underlying conditions were similar to previous findings based on EIP surveillance data collected during 2012–2016. The higher mortality rate associated with candidemia during 2020–2021 likely reflects consequences of the COVID-19 pandemic. Strict implementation of measures to reduce health care–associated bloodstream infections is important for preventing candidemia cases. Health care officials and providers should be vigilant for candidemia as a complication of critical illness. Continued surveillance is needed to monitor for emerging populations at risk for candidemia and changes in antifungal resistance patterns, which can guide antifungal treatment selection.

## Footnotes

* 45 C.F.R. part 46.102(l)(2), 21 C.F.R. part 56; 42 U.S.C. Sect. 241(d); 5 U.S.C. Sect. 552a; 44 U.S.C. Sect. 3501 et seq.
